# A Markov random field model-based approach for differentially expressed gene detection from single-cell RNA-seq data

**DOI:** 10.1093/bib/bbac166

**Published:** 2022-05-05

**Authors:** Biqing Zhu, Hongyu Li, Le Zhang, Sreeganga S Chandra, Hongyu Zhao

**Affiliations:** 1 Program of Computational Biology and Bioinformatics, Yale University, New Haven, CT, 06511, USA; 2 Department of Biostatistics, School of Public Health, Yale University, New Haven, CT, 06511, USA; 3 Department of Neurology, School of Medicine, Yale University, New Haven, CT, 06511, USA; 4 Department of Neuroscience, School of Medicine, Yale University, New Haven, CT, 06511, USA

**Keywords:** scRNA-seq, differential expression, Markov random field, Parkinson’s disease, pseudobulk

## Abstract

The development of single-cell RNA-sequencing (scRNA-seq) technologies has offered insights into complex biological systems at the single-cell resolution. In particular, these techniques facilitate the identifications of genes showing cell-type-specific differential expressions (DE). In this paper, we introduce MARBLES, a novel statistical model for cross-condition DE gene detection from scRNA-seq data. MARBLES employs a Markov Random Field model to borrow information across similar cell types and utilizes cell-type-specific pseudobulk count to account for sample-level variability. Our simulation results showed that MARBLES is more powerful than existing methods to detect DE genes with an appropriate control of false positive rate. Applications of MARBLES to real data identified novel disease-related DE genes and biological pathways from both a single-cell lipopolysaccharide mouse dataset with 24 381 cells and 11 076 genes and a Parkinson’s disease human data set with 76 212 cells and 15 891 genes. Overall, MARBLES is a powerful tool to identify cell-type-specific DE genes across conditions from scRNA-seq data.

## Introduction

Single-cell RNA-sequencing (scRNA-seq) methods have opened up new opportunities in biological and biomedical research [1–3]. Different from traditional bulk RNA-seq technologies [4], scRNA-seq technology can measure gene expression at single-cell resolution to study tissue heterogeneity [5], which facilitates different downstream explorations such as cell-type-specific differential expression (DE) analysis across conditions.

Many methods have been developed for DE analysis. These methods can be broadly divided into three groups, including those designed for bulk RNA-seq data but are also widely applied to scRNA-seq data, those developed specifically for single cell data, and ensemble methods that combine results from different individual tools. The first group includes DESeq2 [6], edgeR [7] and limma-voom [8, 9]. DESeq2 [6] uses gene-specific Empirical Bayes shrinkage estimation for dispersions based on a negative binomial distribution. Similarly, edgeR [7] employs a negative binomial model to explain both biological variability and technical one, and the dispersion is estimated by the empirical Bayes method. Limma-voom [8, 9] estimates the mean-variance trend and incorporates this into the limma pipeline. Methods that are specifically developed for scRNA-seq data include Model-Based Analysis of Single-Cell Transcriptomics (MAST) [10], Monocle2 [11], Single-Cell Differential Expression (SCDE) [12], Statistical Approach for Identifying Differential Distributions in Single-Cell RNA-seq Experiments (scDD) [13] and Discrete Distributional Differential Expression (D3E) [14]. MAST [10] employs a two-part generalized linear model to account for the bimodal distribution of the data and includes the cellular detection rate as a covariate. Monocle2 [11] develops a generalized additive model for DE analysis and introduces the Census algorithm to estimate the relative transcript count. SCDE [12] fits a mixture probabilistic model composed of a Poisson distribution and a negative binomial distribution to account for the drop-outs and the positive mean expressions. scDD [13] utilizes a conjugate Dirichlet process mixture model for the positive expression and a logistic regression for the zero component. D3E [14] is a nonparametric method which uses the Cramer–von Mises test or the Kolmogorov–Smirnov test to identify DE genes. In addition, a recently developed ensemble method scDEA [15] combines 12 bulk and single cell DE methods using a Lancaster’s combined probability test to achieve better performance than each weak learner alone.

Despite the developments of these methods, two issues have not been adequately addressed in these methods to identify DE genes. First, none of the methods considers similarity across cell types, although there is evidence suggesting that similar cell types share many DE genes [16–18], e.g. different types of neurons in brains, or T cells and cytotoxic T cells in lungs. Taking this shared similarity into consideration may boost the power for DE detection. Second, most existing pipelines are limited to comparing cell-type differences but do not take the sample-level differences into consideration when conducting cross condition DE analysis [19, 20]. Given the wide application of single cell technology and the zero-inflated as well as large-scaled scRNA-seq datasets, there is a need to develop a model to address these issues simultaneously in order to better identify DE genes.

**Figure 1:**
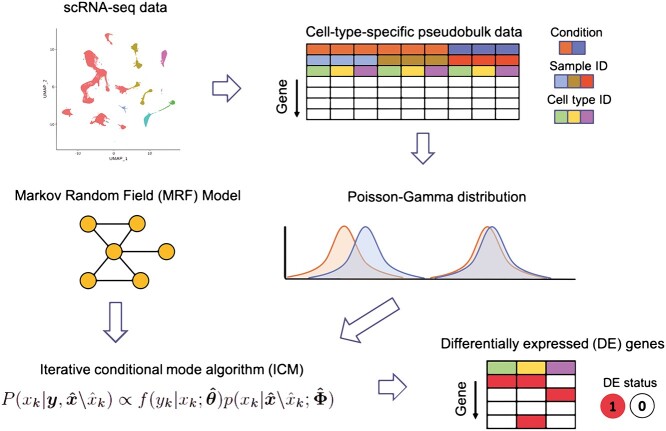
Schematic overview of the MARBLES’ framework. The input of the model is a scRNA-seq dataset and a cell-type relationship network where similar cell types are connected by an edge. Then, the observed scRNA-seq data are converted to cell-type-specific pseudobulk data, and for each gene, a Poisson–Gamma distribution is fitted to the samples in each cell type. An MRF model is implemented based on the cell-type network. Finally, applying ICM, latent states that represent whether genes are differentially expressed can be inferred iteratively.

In this study, we propose MARBLES, a **Mar**kov Random Field (MRF) model-**b**ased approach for differentia**l**ly **e**xpressed gene detection from **s**cRNA-seq data, which can capture cell-type relationships and account for sample variation by modeling cell-type-specific pseudobulk data. We note that MRF-based algorithms have been widely used to model gene relationships in bulk RNA-seq studies as well as genome-wide association studies by incorporating biological pathway information into the analyses [21–24]. They have also been used to model spatial-temporal dependencies [22, 25]. In scRNA-seq analysis, cell-type-specific pseudobulk count is calculated by aggregating all the counts for a specific cell type in one sample, and these pseudobulk data have been used to evaluate the similarity between bulk and imputed scRNA-seq profiles [26], to alleviate plate effects [27] and to identify cell-type-specific DE genes [19]. MARBLES combines a two-group empirical Bayes Poisson–Gamma model [28] to fit the cell-type-specific pseudobulk counts with an MRF model to account for the dependencies among cell types. We have implemented this model using an iterative conditional mode algorithm (ICM) [29] to estimate model parameters and identify cell-type-specific DE genes (Figure 1).

## Methods

### Notations

Given scRNA-seq gene expression profiling data under different conditions, we aim to identify cell-type-specific DE genes. We assume that each gene in each cell type can have two states, labeled as 0 and 1, representing equally expressed (EE) and differentially expressed (DE), respectively. For each gene, we assume that there is a latent state assignment across cell types, which is denoted by $\boldsymbol{x} = (x_1, x_2,\cdots , x_K)$, where $x_k$ is the corresponding state of that gene in cell type $k$ and $K$ denotes the total number of cell types. $x_k$ is 1 if cell type $k$ is DE and 0 otherwise. Also, we define the pseudobulk counts as the sum of the counts of all the cells belonging to a specific cell type in an individual. Let $\boldsymbol{y}_k$ denote the pseudobulk expression level of a gene in cell type $k$, which can be thought of as a realization of a random vector, $\boldsymbol{Y} = (\boldsymbol{Y}_1, \boldsymbol{Y}_2, \cdots , \boldsymbol{Y}_K)$, and $\boldsymbol{y}_k$ itself is also a vector $\boldsymbol{y}_k = (y_{k1},y_{k2},\cdots ,y_{km}; y_{k(m+1)},\cdots , y_{k(m+n)})$, consisting of $m$ individuals under one condition and $n$ individuals for the other condition.

We further assume that given the latent states $\boldsymbol{x} = (x_1, x_2,\cdots , x_K)$, the random variables $\boldsymbol{Y} = (\boldsymbol{Y}_1, \boldsymbol{Y}_2, \cdots , \boldsymbol{Y}_K)$ are conditionally independent and all the $\boldsymbol{Y}_k$ have the same underlying conditional probability distribution $f(\boldsymbol{y}_k|x_k)$ depending only on the latent state $x_k$. And given $\boldsymbol{x}$, the conditional probability of the pseudobulk counts $\boldsymbol{y}$, is

(1) \begin{align*}& l(\boldsymbol{y}|\boldsymbol{x}) = \prod_{k=1}^K f(\boldsymbol{y}_k|x_k). \end{align*}

### Poisson–Gamma model for pseudobulk data

For each gene, we assume that $y_{ki}$ follows a Poisson distribution with mean value $\lambda _k$, where $i$ is the sample index. Therefore, the corresponding density function can be written as

(2) \begin{align*}& f(y_{ki} | \lambda_k) = \frac{\lambda_k^{y_{ki}} e^{-\lambda_k}}{{y_{ki}}!}. \end{align*}

Furthermore, we assume that $\lambda _k$ follows a gamma distribution with shape $\alpha $ and rate $\beta $

(3) \begin{align*}& f(\lambda_k) = \frac{\beta^\alpha \lambda_k^{\alpha - 1} e^{-\beta \lambda_k}}{\Gamma(\alpha)}. \end{align*}

Let $\boldsymbol{\theta } = (\alpha , \beta )$ denote the parameters used to specify these two distributions, so the joint predictive probability for the pseudobulk counts $\boldsymbol{y}_k$ under the same condition is

(4) \begin{align*}& f(\boldsymbol{y}_k) = \int (\prod_{y \in \boldsymbol{y}_k} f(y|\lambda_k)) f(\lambda_k) d\lambda_k. \end{align*}

With these assumptions, we can derive the joint density for the observations from the first condition

(5) \begin{align*}& f(y_{k1},\cdots,y_{km}) = \frac{\beta^\alpha \Gamma((\sum_{j = 1}^{m} y_{kj}) + \alpha)}{(\prod_{j=1}^{m} (y_{kj}!)) \Gamma(\alpha) (m + \beta)^{(\sum_{j=1}^{m} y_{kj}) + \alpha}}, \end{align*}

as well as those from the second condition

(6) \begin{align*} &f(y_{k(m+1)},\cdots,y_{k(m+n)}) \nonumber\\ &= \frac{\beta^\alpha \Gamma((\sum_{j = m + 1}^{m + n} y_{kj}) + \alpha)}{(\prod_{j = m + 1}^{m + n} (y_{kj}!)) \Gamma(\alpha) (n + \beta)^{(\sum_{j = m + 1}^{m + n} y_{kj}) + \alpha}}. \end{align*}

Thus, conditioning on the DE state $x_k$ and $\boldsymbol{\theta }$, we have

(7) \begin{align*} &\quad f(\boldsymbol{y}_k | x_k ; \boldsymbol{\theta}) \nonumber\\ &= [f(y_{k1}, \cdots, y_{km}) f(y_{k(m+1)}, \cdots, y_{kn})]^{x_k} \nonumber\\ &\quad [f(y_{k1}, \cdots, y_{km}, y_{k(m+1)}, \cdots, y_{kn})]^{1 - x_k} \nonumber\\ &= \left[\frac{\beta^{2\alpha} \Gamma((\sum_{j = 1}^{m} y_{kj}) + \alpha) \Gamma((\sum_{j = m + 1}^{m + n} y_{kj}) + \alpha)}{\Gamma(\alpha)^2 (\prod_{j = 1}^{m + n} (y_{kj}!)) (m + \beta)^{\big(\sum_{j = 1}^{m} y_{kj}\big) + \alpha} (n + \beta)^{\big(\sum_{j=m+1}^{m+n} y_{kj}\big) + \alpha}}\right]^{x_k} \nonumber\\ &\quad \left[\frac{\beta^\alpha \Gamma((\sum_{j=1}^{m+n} y_{kj}) + \alpha)}{\Gamma(\alpha) (\prod_{j = 1}^{m+n} (y_{kj}!)) (m+n + \beta)^{(\sum_{j=1}^{m+n} y_{kj}) + \alpha}}\right]^{1-x_k}. \end{align*}

More detailed derivations can be found in Supplementary Texts 1.1. (see Supplementary Data available online at http://bib.oxfordjournals.org/).

Then, the conditional probability of a gene’s pseudobulk across all $K$ cell types has the following form:

(8) \begin{align*}& l(\boldsymbol{y}|\boldsymbol{x};\boldsymbol{\theta}) = \prod_{k=1}^K f(\boldsymbol{y}_k|x_k;\boldsymbol{\theta}). \end{align*}

### MRF Model

A gene’s DE states across cell types are not independent. For example, if a gene is differentially expressed in natural killer cells, it is likely that the gene is also a DE gene in group 1 innate lymphoid cells (ILC1) due to their functional similarities [17, 30]. In order to incorporate such cell-type dependency when conducting DE analysis, we construct an MRF model based on the known cell-type relationship network. In our model, for each gene, the network is represented by an undirected graph $G = \{V, E\}$, where $V$ is the set of nodes representing the cell types and $E$ is the set of edges which correspond to the relationships among cell types. More specifically, for two cell types $k$ and $k^{\prime}$, if they are related, we write $k \sim k^{\prime}$. For a specific cell type $k$, let $N_k = \{k^{\prime}:k \sim k^{\prime} \in E\}$ be the subset of cell types that are linked to cell type $k$. Then, we propose to construct a pairwise interaction MRF model with parameter $(\gamma _0, \gamma _1, \beta )$ for each gene

(9) \begin{align*}& p(\boldsymbol{x}; \gamma_0, \gamma_1, \beta) \propto exp(\gamma_0 n_0 + \gamma_1 n_1 - \beta n_{01}), \end{align*}

where $n_0 = {\sum _{k=1}^K} (1 - x_k)$ denotes the number of cell types at EE, $n_1 = {\sum _{k=1}^K} (x_k)$ represents the number of cell types at DE and $n_{01}$ is the number of edges connecting two cell types with different states. Here, $\gamma _0$ and $\gamma _1$ are free parameters and we do not put any constraints on them. $\beta $ is the parameter that captures the cell-type connections, and we set $\beta $ to be positive in order to penalize neighboring cell types having different states.

Let $\gamma = \gamma _1 - \gamma _0$, and $\boldsymbol{\Phi } = (\gamma , \beta )$, then based on any two state assignments which only differ at cell type $k$, it is easy to derive the conditional probability for cell type $k$, given the states of all the other cell types

(10) \begin{align*}& p(x_k | \boldsymbol{x} \backslash x_k; \boldsymbol{\Phi}) = \frac{exp\{x_k F(x_k;\boldsymbol{\Phi})\}}{exp\{F(x_k; \boldsymbol{\Phi})\} + 1}, \end{align*}

where ‘means other than’, and

(11) \begin{align*}& F(x_k;\boldsymbol{\Phi}) = \gamma - \beta \sum_{k^{\prime} \in N_k} (2x_{k^{\prime}} - 1). \end{align*}

The estimation of $\boldsymbol{\Phi }$ is set to maximize the conditional likelihood based on the ‘coding method’ [31]

(12) \begin{align*}& l(\boldsymbol{x}; \boldsymbol{\Phi}) = \prod_{k=1}^K p(x_k|\boldsymbol{x} \backslash x_k ; \boldsymbol{\Phi}) \nonumber\\ & \qquad = \prod_{k=1}^K \frac{exp\{x_k F(x_k;\boldsymbol{\Phi})\}}{exp\{F(x_k; \boldsymbol{\Phi})\} + 1}. \end{align*}

### Parameter estimation based on ICM

The parameter set $\boldsymbol{\theta }$ for the Poisson–Gamma model and the $\boldsymbol{\Phi }$ for the MRF model need to be estimated simultaneously in order to conduct the inference on the latent states $\boldsymbol{x}$ for the $K$ cell types. Here, we adopt the ICM algorithm proposed by Besag [29] to estimate those parameter sets. For each gene, the algorithm proceeds as follows:

(i) Initialization: Obtain initial estimated states $\boldsymbol{\hat{x}}$ from any established DE method.
(ii) Estimation of $\boldsymbol{\theta }$: Obtain maximum likelihood estimates from $l(\boldsymbol{y}|\boldsymbol{\hat{x}};\boldsymbol{\theta })$, based on Equation 8.
(iii) Estimation of $\boldsymbol{\Phi }$: Maximize the conditional likelihood $l(\boldsymbol{\hat{x}};\boldsymbol{\Phi })$ [see Equation 12] based on the current $\boldsymbol{\hat{x}}$ to obtain $\boldsymbol{\hat{\Phi }}$.
(iv) Update $\boldsymbol{x}$: Perform one round of ICM using the current estimated values of $\boldsymbol{\hat{x}}$, $\boldsymbol{\hat{\theta }}$ and $\boldsymbol{\hat{\Phi }}$ to get an updated $\boldsymbol{x}$. In particular, we choose $x_k$ = 1 or $x_k$ = 0, whichever maximizes the conditional probability
  (13) \begin{align*}& P(x_k|\boldsymbol{y},\boldsymbol{\hat{x}} \backslash \hat{x}_k) \propto f(y_k | x_k; \boldsymbol{\hat{\theta}}) p(x_k| \boldsymbol{\hat{x}} \backslash \hat{x}_k;\boldsymbol{\hat{\Phi}}). \end{align*}
(v) Repeat steps (2)–(4) until convergence or for a fixed number of iterations.

The resulting $\boldsymbol{\hat{x}}$ can be seen as an approximate of the true latent states, and more technical details can be found in Supplementary Texts 1.2. (see Supplementary Data available online at http://bib.oxfordjournals.org/).

## Simulation Studies

### Simulation setup

A simulation study was performed to evaluate the performance of MARBLES. To simulate the cell-type relationship network, we set the number of cell types to be six, and randomly selected $50\%$ of the cell type pairs to be connected. Both the number of cell types and connectivity were selected to match the Parkinson’s disease (PD) data in our real data application in section 4.3, which is a single-nucleus RNA-seq (snRNA-seq) dataset of the brain cortex tissue consisting of six cell types from 12 individuals divided into PD and healthy control (HC) groups. Then, to simulate the latent states $\boldsymbol{X}$ for each gene across cell types, we initialized a random set of cell types to be DE and the rest of the cell types to be EE, resulting in $\boldsymbol{X_0}$. Next, based on the cell-type network structure, starting from $\boldsymbol{X_0}$, we performed Gibbs sampling five times to get the final latent states $\boldsymbol{X}$. In each cycle of the Gibbs sampling, the latent states were updated entrywise according to Equation (10). Here, we set $\boldsymbol{\Phi } = (-10, 11)$ which were the modes of the distributions of the PD data parameter estimation.

Next, given $\boldsymbol{X}$, we simulated the count data. To explicitly take into account the individual effects as well as the cell-type effects, we adopted the simulation model from the *muscat* [19] and incorporated our cell-type relationship network, yielding a new simulation model which consists of the following steps:

(i) Estimation of negative binomial (NB) parameters based on the reference PD data. To better set the baseline, we only chose the six HC individuals as our reference for simulation. Then, the cell-type- and sample-specific means, dispersion and the library size for the NB distribution were estimated from the reference dataset.
(ii) Sampling count data based on the cell-type relationship network. For each gene and cell type, we assigned the latent states (DE or EE) according to $\boldsymbol{X}$. If a gene in a cell type was DE, we sampled a log fold change (logFC) from a Gamma distribution with $\alpha = 4$ and $\beta = 4 / \tau $, where $\tau $ was the average logFC across genes and cell types. For a DE cell type in a specific gene, it had equal probability of being up-regulated or down-regulated. For EE cell types, counts from the two conditions were sampled from the same mean. And for DE cell types, the mean of a random condition was multiplied by the resulting fold change. Thus, the baseline multi-cell type, multi-sample count data can be sampled from the resulting distributions. More details can be found in their original paper.

### Model benchmarking comparison

The number of genes $G$ was set to be 1000. To test the model robustness and also the ability to detect DE genes in single cell data at different scales or levels of complexity, we varied three parameters $n_c$ (Scenario 1), $n_s$ (Scenario 2) and $\tau $ (Scenario 3), where $n_c$ is the number of cells of each cell type in each sample, $n_s$ represents the number of samples in each condition and $\tau $ is the average logFC mentioned in the previous section. In Scenario 1, $n_c$ ranges from 100 to 1000, $\tau $ is chosen to be 2 and $n_s$ be 4. In Scenario 2, we chose $n_s$ to be between 3 and 12, $n_c$ is fixed to be 150 and $\tau $ to be 2. In Scenario 3, $\tau $ varies from 1.2 to 3.8, $n_c$ is 150 and $n_s$ is 4. Currently, there are no gold standard methods for detecting DE genes [32, 33], and the agreement among those most widely used algorithms is relatively low [33]. We initialized our models using (i) three commonly used bulk methods, DESeq2 [6], edgeR [7] as well as limma-voom [8, 9], (ii) the ensemble method scDEA [15] and (iii) the individual single cell methods considered in scDEA, including BPSC [34], DEsingle [35], monocle [36], scDD [13], T-test [37], Wilcoxon test [38], SeuratBimod [39, 40] and zingeR.edgeR [41]. In addition, to test the model robustness against different initializations $\boldsymbol{\hat{X}}$, we also initialized MARBLES with (iv) random states. For method sets (i) and (iv), we ran simulation under all three scenarios and repeated the simulation 50 times, whereas for the method sets (ii) and (iii), due to the runtime and memory constraints, we only tested them on Scenario 1 with five repeats. The reason we specifically chose the method set (i) is that these methods are all tailored for bulk RNA-seq data and thus fit our model assumption where we modeled the distribution of the cell-type-specific pseudobulk data to account for the sample variation. Therefore, this approach is adequate to benchmark the performance of our method.

As for the DE gene detection threshold, we set it to be the locally Benjamini and Hochberg (BH) adjusted *P*-value less than 0.05, and abs(logFC) $> \tau / 2$. Here, locally means the multiple testing correction was performed on each of the cell-type-level test ($n = G$), in order to be less conservative and have a higher sensitivity [19]. Since for our model, the outputs are the latent states (DE or EE) instead of the *P*-values, we only applied the second threshold as our criteria for MARBLES.

## Results

### Simulation studies

**Figure 2:**
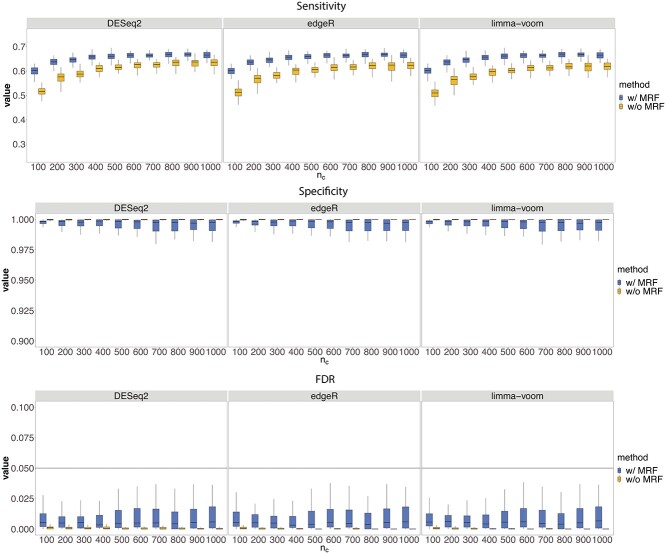
Simulation results for method set (i) under Scenario 1. The sensitivity, specificity and FDR are plotted under different $n_c$s for DESesq2, edgeR, limma-voom alone (w/o MRF) or for the MARBLES model initialized with those methods (w/ MRF).

The simulation results for method set (i) Scenario 1 are shown in Figure 2, those for Scenarios 2 and 3 are shown in Supplementary Figures 1 and 2 (see Supplementary Data available online at http://bib.oxfordjournals.org/). In each Scenario, we compared the performance of DESeq2, edgeR and limma-voom alone (w/o MRF), against MARBLES initialized with those methods (w/ MRF), in terms of sensitivity, specificity and false discovery rate (FDR). In general, the three methods had similar performance in different scenarios, and so did our model when initializing with those methods’ estimates. In Scenario 1 (Figure 2), it is not surprising that as $n_c$ increases the performance of all the models improves, but MARBLES consistently shows much higher sensitivity, comparable specificity and well-controlled FDR (less than 0.05). Additionally, the model was very robust when for different $n_s$ and had the best performance (Supplementary Figure 1, see Supplementary Data available online at http://bib.oxfordjournals.org/). In terms of $\tau $, simulation results show that MARBLES was comparable with the other three methods when $\tau $ was around 1.6, and continued to improve when $\tau $ increased, which was within the range of the current scRNA-seq datasets (Supplementary Figure 2, see Supplementary Data available online at http://bib.oxfordjournals.org/). The results for MARBLES initialized with method sets (ii) and (iii) are in Supplementary Figures 3 and 4 (see Supplementary Data available online at http://bib.oxfordjournals.org/), respectively, where MARBLES also outperformed all the other methods in terms of sensitivity, had a comparable specificity and well-controlled FDR. Additionally, the random initialized model results are shown in Supplementary Figure 5 (see Supplementary Data available online at http://bib.oxfordjournals.org/), and although comparable sensitivity can be obtained using randomized $\boldsymbol{\hat{X}}$, the standard deviations of specificity and FDR are much larger under all three settings, which shows the benefit of using the outputs from other DE methods for MARBLES to get more stable results.

### Application to LPS mouse cortex data

**Figure 3:**
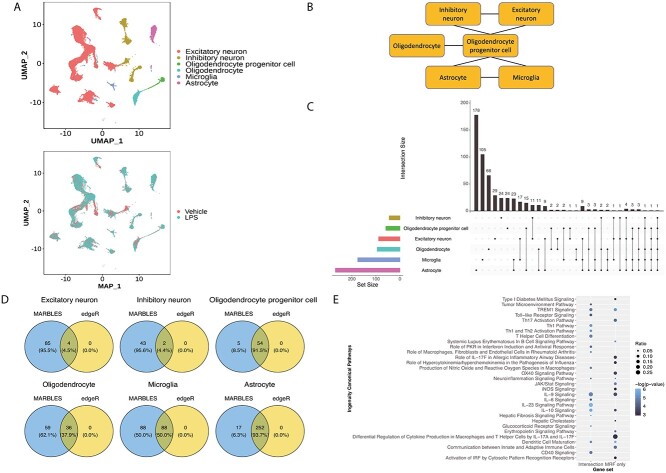
edgeR-MARBLES results on the LPS mouse cortex data. (A) The UMAP plot of the LPS mouse dataset colored by cell type (top) and treatment condition (bottom). (B) The cell-type relationship network among these cell types which was built based on domain knowledge. (C) The total number of DE genes identified by MARBLES for each cell type (horizontal bar plots) and the overlap among cell types (vertical bar plots and lines). (D) Venn diagrams showing the gene sets identified by edgeR alone or MARBLES for each cell type. (E) The top IPA pathways of the DE genes in microglia identified by both edgeR and MARBLES (Intersection) or MARBLES only (MRF only).

The performance of MARBLES was first evaluated on a mouse cortex snRNA-seq dataset in Crowell *et al.* [19]. LPS is known to cause neuro-inflammation and neuronal cell death in the brain [42, 43]. This dataset has already been preprocessed as well as annotated, and contains four control (Vehicle) and four LPS-treated mice (Figure 3A bottom). Since we wanted to focus on neurons and glial cells, we only selected excitatory neurons, inhibitory neurons, astrocytes, microglia, oligodendrocyte progenitor cells (OPCs) and oligodendrocytes for downstream analyses (Figure 3A top), resulting in 11 076 genes and 24 381 cells. The cell-type relationship was determined by domain knowledge to represent the similarity and cell lineage (Figure 3B). After aggregating the data into cell-type-specific pseudobulk, we applied MARBLES initialized with both the edgeR (edgeR-MARBLES) and the scDEA (scDEA-MARBLES) results. Due to the high demand of memory and runtime for scDEA (Figure 5), only 25% of the cells from each cell type were subsampled to feed into the scDEA algorithm. Here, the threshold for DE gene detection was set to be abs(logFC) > 1, and the gene expression in a specific cell type was larger than the 40th percentile of the cell-type-specific gene mean expression across samples of all genes and all cell types. As for edgeR and scDEA, similar to the simulation settings, an additional requirement is that the BH adjusted *P*-value being less than 0.05. The distribution of the estimated parameter $\boldsymbol{\Phi }$ for the edgeR-MARBLES model across all genes is shown in Supplementary Figure 6A (see Supplementary Data available online at http://bib.oxfordjournals.org/). edgeR-MARBLES and scDEA-MARBLES identified 554 and 582 DE genes in at least one cell type, whereas edgeR and scDEA only detected 373 and 527 ones, respectively. Figure 3C is the UpSet [44] plot showing the total number of genes (horizontal bars), and unique DE genes for each cell type as well as the overlap DE genes across cell types (vertical bars and lines) identified by edgeR-MARBLES. Astrocytes had the largest number of DE genes, followed by microglia, and neurons had relatively small sets of DE genes. Also, most genes were cell-type specific, but the cell types within glial cells or neurons also share some genes, as expected. We further investigated the directions of the shared DE genes between similar cell types to see if edgeR-MARBLES can indeed borrow information across these cell types to find biologically meaningful genes, and we found that all the genes shared the same logFC direction (Supplementary Figure 6B, see Supplementary Data available online at http://bib.oxfordjournals.org/), although the algorithm itself does not impose the constraint of the DE direction. The exact logFC can be found in Supplementary Table 1 (see Supplementary Data available online at http://bib.oxfordjournals.org/). In addition, to test model robustness and to consider the situation where the true cell-type relationships are unknown, four alternative cell-type networks were constructed (Supplementary Texts 1.3, Supplementary Figure 7A–D, see Supplementary Data available online at http://bib.oxfordjournals.org/), based on which we ran edgeR-MARBLES. Overall, the numbers of DE genes identified by the four alternative models were similar to the ones returned by the main model (Supplementary Figure 7E, see Supplementary Data available online at http://bib.oxfordjournals.org/). Note that in the fourth alternative network (Supplementary Figure 7D, see Supplementary Data available online at http://bib.oxfordjournals.org/), oligodendrocyte is disconnected from the rest of the cell types, which may explain why this model identified the smallest set of DE genes for this cell type (Supplementary Figure 7I, see Supplementary Data available online at http://bib.oxfordjournals.org/). Nevertheless, the cell-type-specific DE genes resulted from the five models are quite similar for other cell types (Supplementary Figure 7F–H, J-K, see Supplementary Data available online at http://bib.oxfordjournals.org/), demonstrating MARBLES’s robustness to network misspecification.

Next, we clustered all the edgeR-MARBLES DE genes from the main model according to their cell-type-specific logFC using the consensus clustering through M3C [45]. Three clusters were returned and most of the genes in cluster 3 were upregulated in LPS mice especially in glial cells (Supplementary Figure 6C, see Supplementary Data available online at http://bib.oxfordjournals.org/) and related to immune responses similar to previous studies [19] (Supplementary Figure 6D, see Supplementary Data available online at http://bib.oxfordjournals.org/). Then, we compared the number of DE genes identified by edgeR and edgeR-MARBLES, with edgeR in yellow and edgeR-MARBLES in blue. Utilizing cell-type relationship network, edgeR-MARBLES could detect more genes than edgeR for all the cell types (Figure 3D), and similar results were also found between scDEA and scDEA-MARBLES (Supplementary Figure 6A, see Supplementary Data available online at http://bib.oxfordjournals.org/). The complete list of DE genes can be found in Supplementary Table 2 (see Supplementary Data available online at http://bib.oxfordjournals.org/).

Furthermore, to gain more biological insights from the DE genes, we performed canonical pathway analysis using the Ingenuity Pathway Analysis (IPA) [46] for each cell type based on either the overlap genes between edgeR/scDEA and MARBLES (Intersection), or the novel genes identified by MARBLES alone (MRF only). The pathways with BH corrected *P*-value less than 0.05 were considered significant. The results for microglia are shown in Figure 3E and Supplementary Figure 6B (see Supplementary Data available online at http://bib.oxfordjournals.org/) as an example. For the edgeR/MARBLES comparison, not surprisingly, using the intersection genes, many immune-related pathways were identified [47–49]. For example, triggering receptor expressed on myeloid cells 1 (TREM1), which is related to microglial maladaptive responses, shows a significant increase following the induced brain inflammation cause by LPS [50, 51], and also LPS can be a stimulus for microglial activation, causing the elevated expression of the toll-like receptors (TLR) [52, 53], the enhanced secretion of the neural damage correlated proinflammatory molecule interleukin 6 (IL-6) [54, 55]. Besides that, our model identified an additional set of pathways, of which, interferon regulatory factor (IRF) activation in microglia is critical for inflammatory response mediation in the brain [56, 57], high amount of nitric oxide synthase (iNOS) can be induced in LPS-stimulated microglia [58, 59] and JAK/STAT signaling is one of the pathways that can induce microglia activation upon LPS stimulation [54, 60]. Likewise, scDEA-MARBLES identified extra immune-related pathways, such as Th17 activation pathway [61], JAK/STAT signaling and IL-13 signaling pathway [62], all of which have been found to be associated with neuroinflammation. The pathways inferred from both sets of genes for each cell type are provided in Supplementary Table 3 (see Supplementary Data available online at http://bib.oxfordjournals.org/). Taken together, our model can not only find genes that are in agreement with the establish method but also detect novel genes which are related to biologically meaningful pathways.

### Application to the PD human prefrontal cortex data

**Figure 4:**
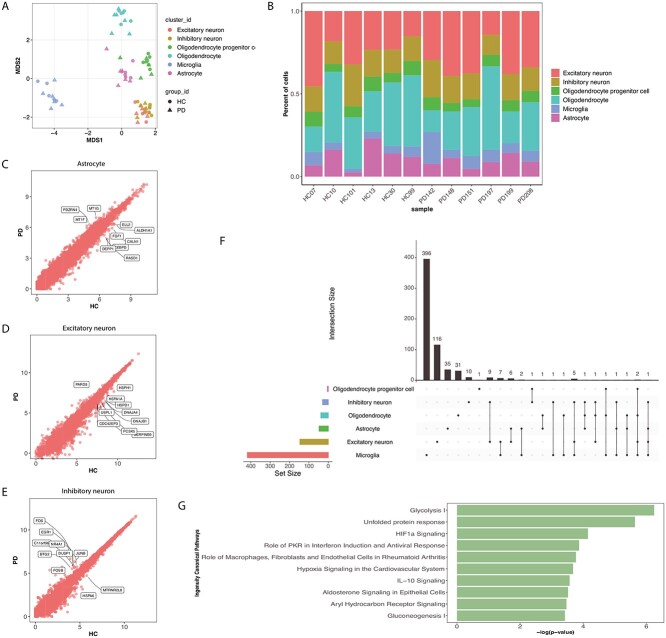
edgeR-MARBLES results on the PD human prefrontal cortex data. (A) The MDS plot of the cell-type-specific pseudobulk-level PD data colored by cell type and shaped by disease condition. (B) Cell-type proportions in each individual. (C–E) Scatter plots of the pseudobulk-level mean expression of each gene for PD and HC in astrocytes (C), excitatory neurons (D) and inhibitory neurons (E). Top 10 DE genes based on mean expression value of the pseudobulk data in log scale are shown for astrocytes and excitatory neurons, and OPCs only renders five DE genes. (F) The total number of DE genes identified by MARBLES for each cell type (horizontal bar plots) and the overlap among cell types (vertical bar plots and lines). (G) The top ten microglia IPA pathways of the DE genes identified by MARBLES.

Finally, we applied our model to a single cell dataset containing post-mortem human brain tissue from the prefrontal cortex of six PD patients (denoted by PD142, PD148, PD151, PD197, PD199 and PD208) and six healthy controls (HC) (denoted by HC07, HC10, HC101, HC13, HC30 and HC99) in a recent study [18]. We filtered out T cells and endothelial cells which only made up 1.13% of the total population (Figure 4A and B, Supplementary Figure 9A, see Supplementary Data available online at http://bib.oxfordjournals.org/), resulting in 15 891 genes and 76 212 cells. Then, we applied the same cell-type network (Figure 3B) to conduct the MARBLES analysis. Since edgeR resulted in no DE genes, we initialized our edgeR-MARBLES model with all EE states and identified 630 DE genes expressed in at least one cell type. On the other hand, scDEA (25% subsampling) and scDEA-MARBLES discovered 511 and 631, respectively (Supplementary Figure 9D, see Supplementary Data available online at http://bib.oxfordjournals.org/). Supplementary Figure 9B and C (see Supplementary Data available online at http://bib.oxfordjournals.org/) show the inferred $\boldsymbol{\Phi }$ parameter distribution of the two models, and the complete set of genes identified for both models can be found in Supplementary Table 4 (see Supplementary Data available online at http://bib.oxfordjournals.org/). The edgeR-MARBLES results are shown in Figure 4F, where microglia had the most DE genes, and the two types of neurons shared the largest number of genes. Similarly, we looked into the logFC direction of the DE genes between related cell types and found that most of the genes were of the same direction (Supplementary Figure 9E, see Supplementary Data available online at http://bib.oxfordjournals.org/), and the exact logFC can be found in Supplementary Table 5 (see Supplementary Data available online at http://bib.oxfordjournals.org/). Then, we examined the DE genes by plotting the mean of the PD pseudobulk expression against those for the HC individuals for each gene in each cell type (Figure 4C–E, Supplementary Figure 9F–H, see Supplementary Data available online at http://bib.oxfordjournals.org/) and found that many of them are associated with PD based on previous studies. For example, metallothioneins 1G (MT1G) was found in PD frontal cortex and expressed by astrocytes to protect neurons [63, 64]. Also, heat shock response genes HSPA1A were downregulated in PD patients neurons [18, 65], and our results suggest that they are also DE genes in OPCs and oligodentrocytes, but upregulated. Morevoer, dual specificity phosphatase 1 (DUSP1) was shown to be upregulated in PD to overcome neuron damage [66, 67].

**Figure 5:**
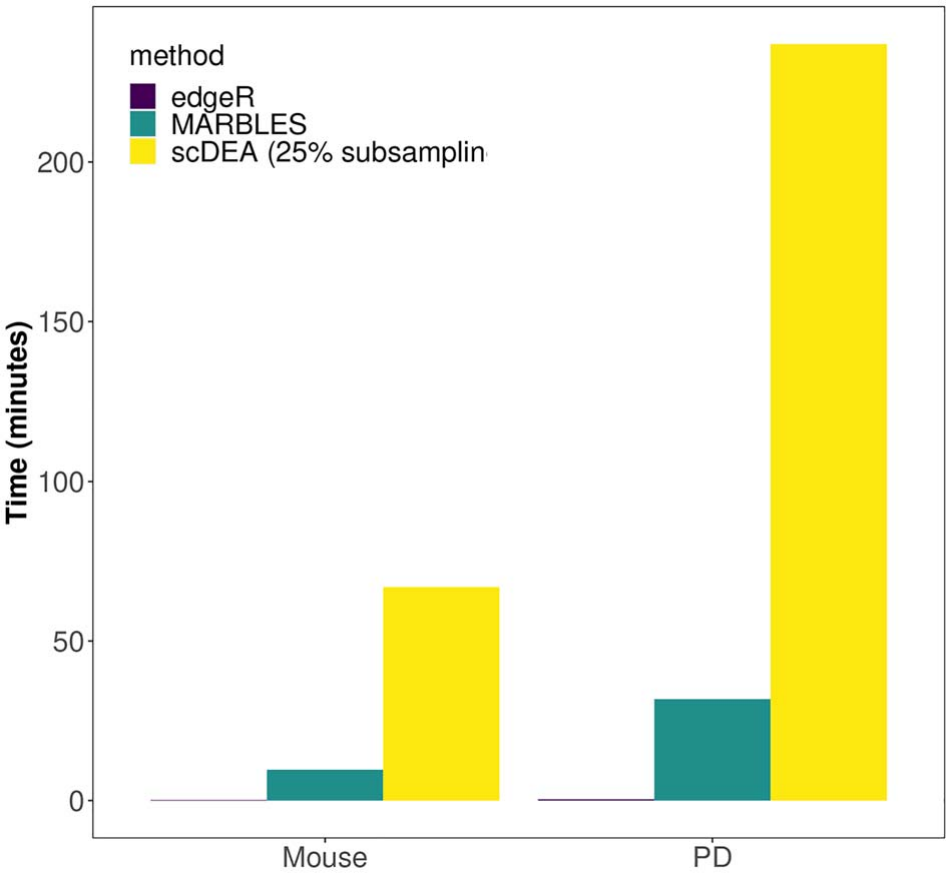
Real data runtime comparison.

Similarly, we carried out the IPA analysis to discover if any known or novel pathways were enriched. Employing the same filtering criteria, we were able to identify 23 pathways for microglia (Supplementary Table 6, see Supplementary Data available online at http://bib.oxfordjournals.org/) and no pathways for other cell types, and Figure 4G shows the top 10 pathways. For instance, studies have shown that unfolded protein response signaling is upregulated in microglia in several neurodegenerative diseases [68, 69]. As expected, the peripheral concentrations of Interleukin 10 (IL-10) was found higher in PD patients [70, 71]. Also, our method indicates that glycolysis, which was reported to be upregulated in microglial cells in Alzheimer’s disease patients to regulate the innate inflammatory response [72, 73], could also be activated in response to PD pathology.

## Discussion

In this paper, we developed a powerful method MARBLES to conduct DE analysis between conditions for scRNA-seq data, by borrowing information across cell types using the MRF. There are two key differences between our model and another MRF method recently developed by us [21]. The first one is that instead of modeling the distribution of the statistics such as *P*-values rendered from other methods, we directly captured the signals in the data using a Poisson–gamma distribution. And the other is that we modeled all the genes instead of just highly variable ones which may introduce method-specific biases [1, 74, 75]. For the DE state inference, we implemented the ICM algorithm, the initialization of which are the results from existing and well-known methods, indicating that MARBLES can not only integrate the outputs from other methods but also extract additional information by directly modeling the pseudobulk data.

Simulation results show that our method can achieve a high statistical power compared with the other well-known methods and is able to simultaneously control the FDR. Also, MARBLES shows robustness to the number of samples and cells, as well as the logFC. Results from the LPS data analysis suggest that our method is capable of finding the same gene sets as the established methods and can also identify novel genes and pathways that are related to the biological problems that are being studied. In addition, the results from the four alternative cell-type networks suggest that MARBLES is robust to network misspecification and can still identify meaningful genes by constructing affinity/distance-based networks when the cell-type relationship of the dataset being studied is unknown. Meanwhile, for the PD data, one possible reason why the logFC of some of the shared DE genes between cell type pairs are not the same is that microglia are immune cells in the brain, whereas astrocytes and oligodendrocytes are supportive cells, so the impact of the PD pathology on these cell types is not the same. Besides, edgeR could find no DE gene, which might be caused by two factors. The first one is that the differences between conditions in mice are more significant than in humans since the neural inflammation in mice is induced, whereas the cause of PD is more complicated and is still an ongoing research [76, 77]. In addition, although the dataset contains many cells, the number of individuals might not be sufficient for those methods to conduct DE gene analysis. Therefore, given the fact that our method has proven to be reliable and more powerful in both simulations and the LPS data application, the genes found by MARBLES in the PD data are highly likely to be PD-related genes and potential drug targets. Additionally, MARBLES achieved much reasonable runtimes than scDEA for both datasets, although due to the scDEA’s high memory and runtime demand, we only subsampled 25% cells from each cell type for both datasets to run the algorithm.

Future works can focus on three aspects. The first one is that the gene–gene relationship network could be incorporated into this framework to capture the biological pathway information [21, 23, 25]. Additionally, MARBLES can include weights to increase the network resolution. Specifically, if we have several subtypes under a specific cell type, the edges within the subtypes should have larger weights than the edges connecting different cell types. Finally, MARBLES could be extended to have more DE states to include the DE directions, which means that the $n_1$ could be devided into $n_+$ and $n_-$, representing the upregulated and downregulated genes, respectively.

**Key Points**

- We propose MARBLES, a **Mar**kov Random Field model-**b**ased approach for differentia**l**ly **e**xpressed gene detection from **s**cRNA-seq data.
- The method can capture cell-type relationships and account for sample variation by modeling cell-type-specific pseudobulk data.
- Simulation results showed that MARBLES is more powerful than existing methods and applications to real-data identified novel disease-related DE genes and biological pathways from two scRNA-seq datasets.

## Supplementary Material

MARBLES_supp_bbac166

supplementary_table_1_bbac166

supplementary_table_2_bbac166

supplementary_table_3_bbac166

supplementary_table_4_bbac166

supplementary_table_5_bbac166

supplementary_table_6_bbac166

supplementary_bbac166

## Data Availability

The IPF mouse data can be downloaded from the R package muscData, and the PD human data are available at XX. The MARBLES was implemented with R and is freely available at https://github.com/biqing-zhu/MARBLES.

## Author Biographies

**Biqing Zhu** is a PhD student from the Program of Computational Biology and Bioinformatics at Yale University. Her research interests focus on single cell RNA sequencing and multi-omics methodology development.

**Hongyu Li** is pursuing his PhD degree in the Department of Biostatistics at Yale University. His research interests include single cell RNA sequencing and methylation.

**Le Zhang** is an Assistant Professor in the Department of Neurology at Yale School of Medicine. Her research focuses on the immune responses of the central nervous system in neurodegenerative diseases, including Parkinson’s disease, Alzheimer’s disease, HIV-associated dementia and Multiple sclerosis, using cutting-edge single cell technologies.

**Sreeganga S. Chandra** is an Associate Professor in the Departments of Neurology and Neuroscience at Yale University. Her lab explores two related themes, those of synapse loss and neurodegeneration.

**Hongyu Zhao** is the Ira V. Hiscock Professor of Biostatistics and Professor of Statistics and Data Science and Genetics at Yale University. His research interests are the developments and applications of novel statistical methods to address scientific questions in genetics, molecular biology, drug developments and precision medicine.
