# Supplementary material for: A Markov random field model-based approach for differentially expressed gene detection from single-cell RNA-seq data
Source: Brief Bioinform. 2022 May 5;23(5):bbac166. doi: 10.1093/bib/bbac166 (PMC9487630; doi:10.1093/bib/bbac166)
Supplement: MARBLES_supp_bbac166 [file marbles_supp_bbac166.pdf]

1. Supplementary Texts

1.1. Derivation of the conditional distribution of pseudobulk expression

If the differential expression state x_k is 1 (DE), we assume the m individuals from the first condition and the n individuals from the second condition are from two distributions, specifically,

$$f(y_{k1},\cdots,y_{km})f(y_{k(m+1)},\cdots,y_{kn}) = \frac{\beta^{2\alpha}\Gamma((\sum_{j=1}^{m} y_{kj})+\alpha)\Gamma((\sum_{j=m+1}^{m+n} y_{kj})+\alpha)}{\Gamma(\alpha)^2(\prod_{j=1}^{m+n}(y_{kj}!))(m+\beta)^{(\sum_{j=1}^{m} y_{kj})+\alpha}(n+\beta)^{(\sum_{j=m+1}^{m+n} y_{kj})+\alpha}}, \quad (1)$$

and if x_k is 0 (EE), we assume the $m+n$ individuals are from the same distribution, specified by,

$$f(y_{k1},\cdots,y_{km},y_{k(m+1)},\cdots,y_{kn}) = [\frac{\beta^{\alpha}\Gamma((\sum_{j=1}^{m+n} y_{kj})+\alpha)}{\Gamma(\alpha)(\prod_{j=1}^{m+n}(y_{kj}!))(m+n+\beta)^{(\sum_{j=1}^{m+n} y_{kj})+\alpha}}]^{1-x_k}, \quad (2)$$

which shares the same form as the Equation 5 and Equation 6 in the main text.

So the distribution of the observations conditioned on x_k and $\boldsymbol{\theta}$ can be modeled as

$$\begin{aligned} & f(\boldsymbol{y}_k|x_k;\boldsymbol{\theta}) \\ =& [f(y_{k1},\cdots,y_{km})f(y_{k(m+1)},\cdots,y_{kn})]^{x_k} \\ & [f(y_{k1},\cdots,y_{km},y_{k(m+1)},\cdots,y_{kn})]^{1-x_k} \\ =& [\frac{\beta^{2\alpha}\Gamma((\sum_{j=1}^{m} y_{kj})+\alpha)\Gamma((\sum_{j=m+1}^{m+n} y_{kj})+\alpha)}{\Gamma(\alpha)^2(\prod_{j=1}^{m+n}(y_{kj}!))(m+\beta)^{(\sum_{j=1}^{m} y_{kj})+\alpha}(n+\beta)^{(\sum_{j=m+1}^{m+n} y_{kj})+\alpha}}]^{x_k} \\ & [\frac{\beta^{\alpha}\Gamma((\sum_{j=1}^{m+n} y_{kj})+\alpha)}{\Gamma(\alpha)(\prod_{j=1}^{m+n}(y_{kj}!))(m+n+\beta)^{(\sum_{j=1}^{m+n} y_{kj})+\alpha}}]^{1-x_k}. \end{aligned} \quad (3)$$

1.2. Technical details on the parameter estimation algorithm

The maximum likelihood estimation of $\boldsymbol{\theta}$ and $\boldsymbol{\Phi}$ based on the Equation 8 and Equation 12 from the main text was carried out by the R function optim. The convergence conditions of the iterative algorithm are (1) the latent state $\boldsymbol{x}$ does not change, (2) the sum of the differences between the current $\boldsymbol{\theta}$ and $\boldsymbol{\theta}$ from the previous iteration is less than 10^{-4}, and (3) the sum of the differences between the current $\boldsymbol{\Phi}$ and $\boldsymbol{\Phi}$ from the previous iteration is less than 10^{-4}.

1.3. Construction details on alternative cell type networks

Four alternative cell type networks were constructed. The first 50 PCs were computed based on the top 2,000 highly variable genes. Then for the first network (Supplementary Figure 7A), the Euclidean distances among the mean centers of the cell types were calculated and then converted to affinities through a Gaussian kernel, then the two cell types were considered connected in the final network if the affinity between them was larger than 0.8. The second network (Supplementary Figure 7B) was constructed under the same way, except that the median centers were computed instead of the mean centers. For the third and forth networks (Supplementary Figure 7C, D), two cell types were connected if their Euclidean distance between the mean centers or median centers was less than 17.

2. Supplementary Figures

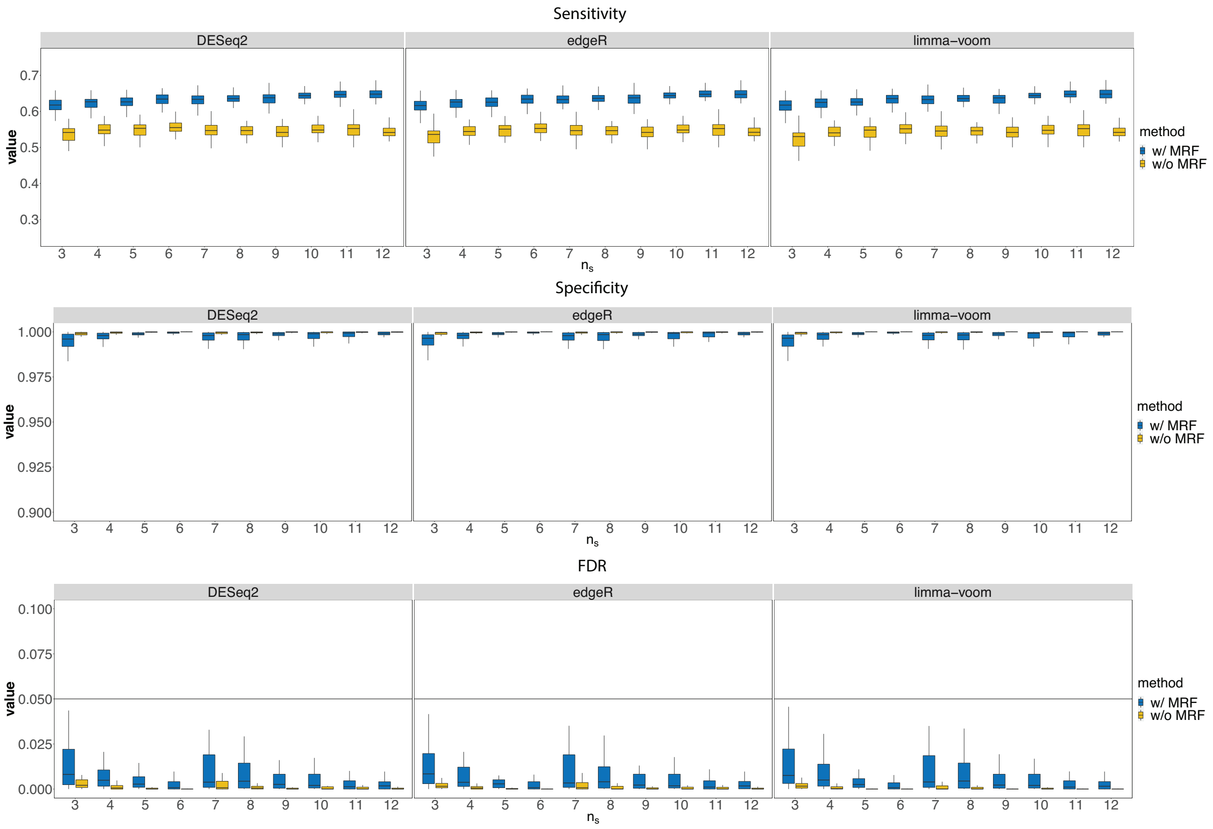

Supplementary Figure 1. Simulation results for method set (1) under Scenario 2. The sensitivity, specificity, and FDR are plotted under different n_ss for DESesq2, edgeR, limma-voom alone (w/o MRF), or for the MRF model initialized with those methods (w/ MRF).

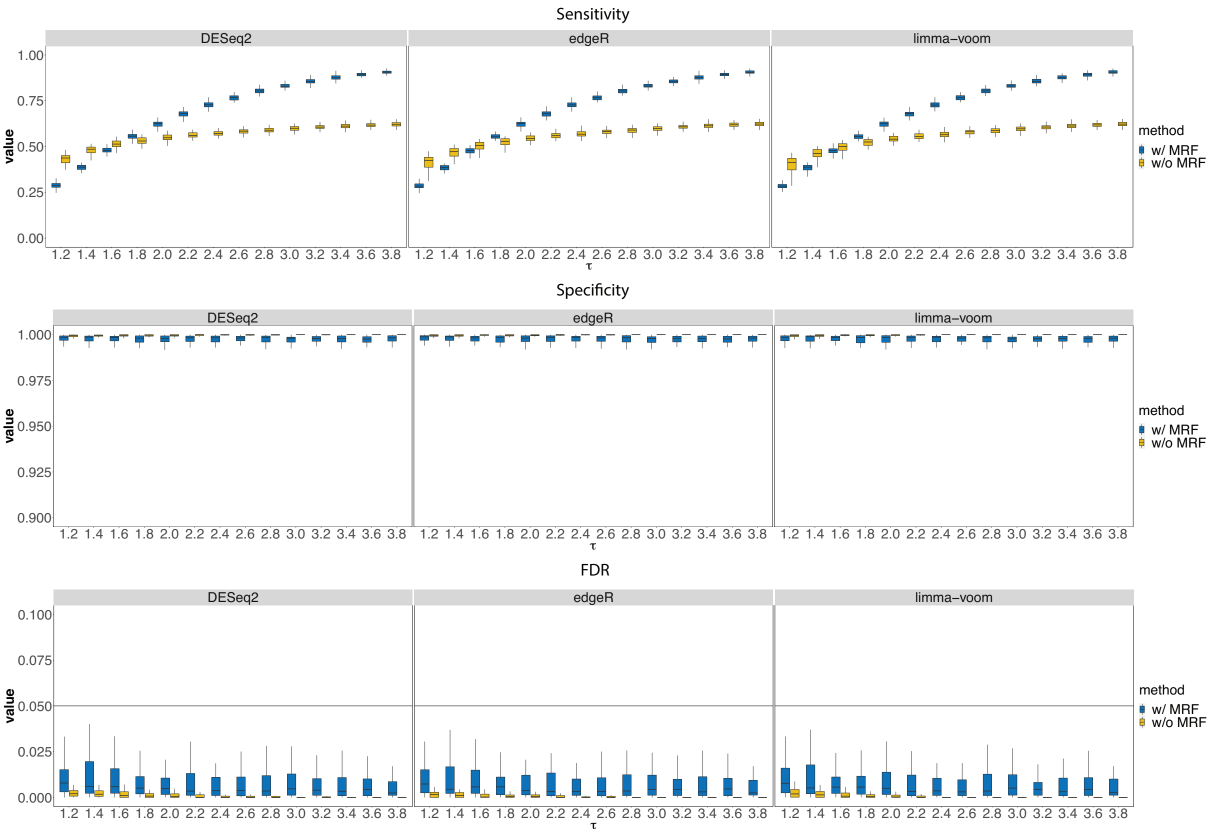

Supplementary Figure 2. Simulation results for method set (1) under Scenario 3. The sensitivity, specificity, and FDR are plotted under different τs for DESesq2, edgeR, limma-voom alone (w/o MRF), or for the MRF model initialized with those methods (w/ MRF).

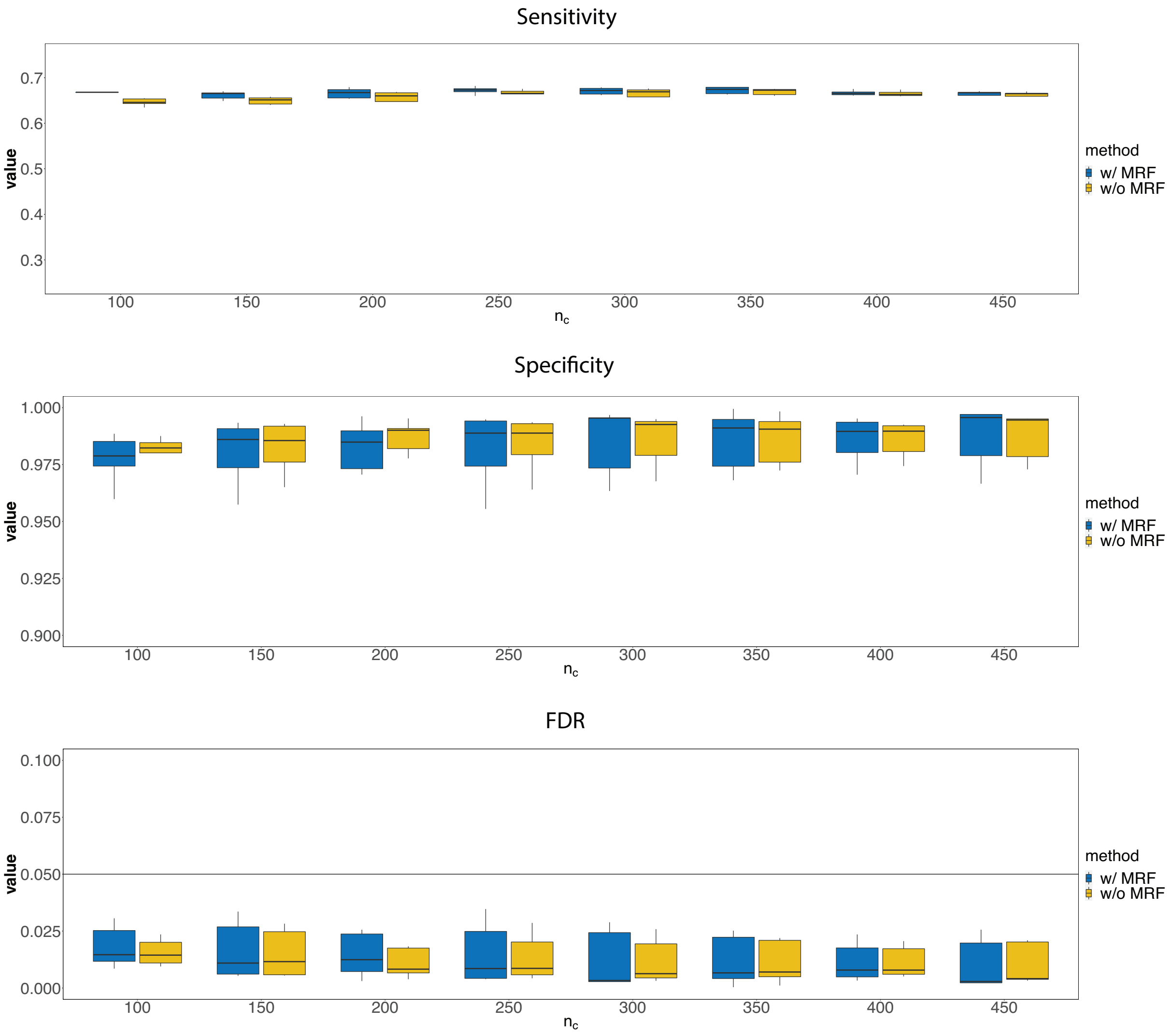

Supplementary Figure 3. Simulation results for scDEA under under Scenario 1. The sensitivity (A), specificity (B), and FDR (C) for different n_c.

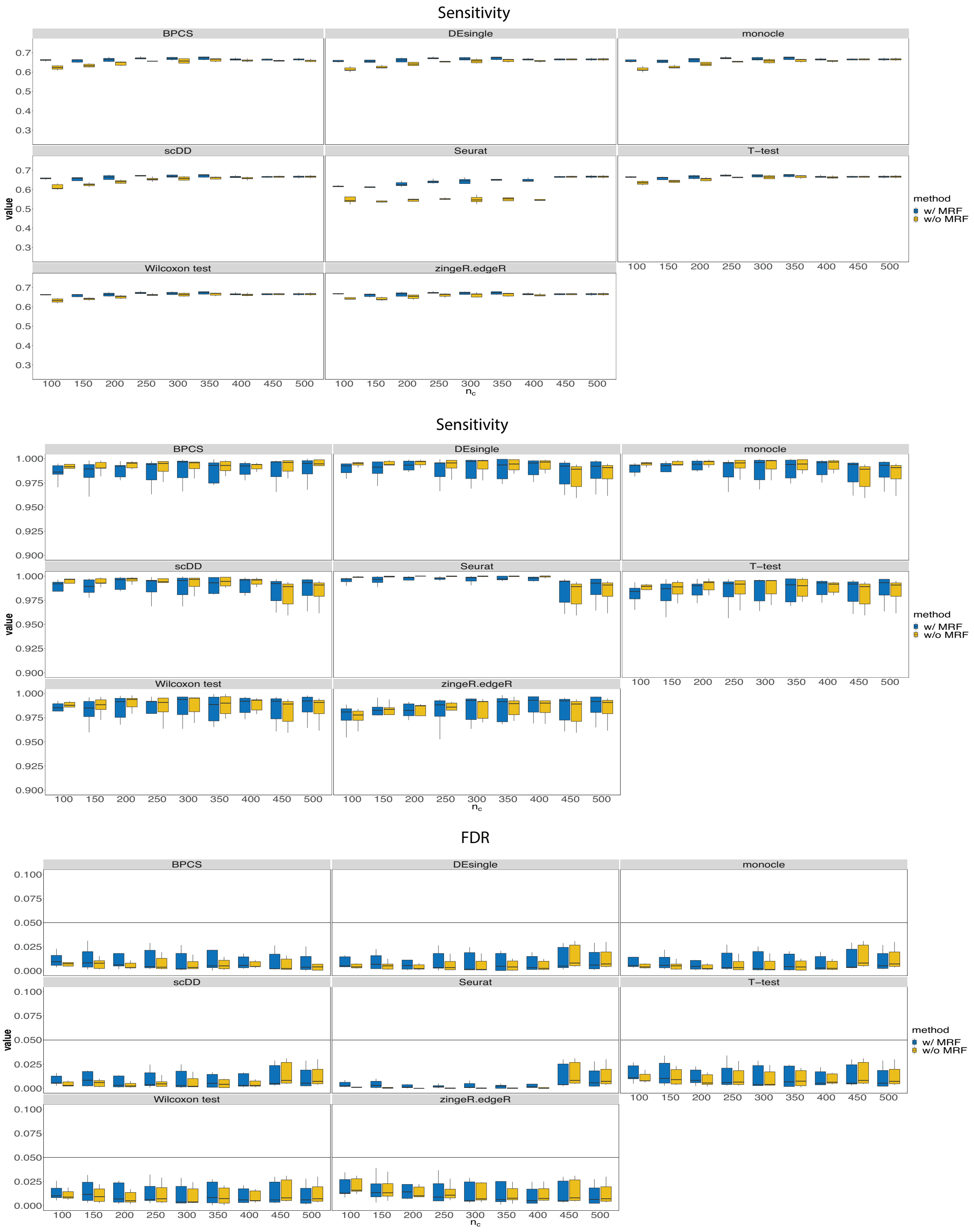

Supplementary Figure 4. Simulation results for method set (3) under under Scenario 1. The sensitivity (A), specificity (B), and FDR (C) for different n_c.

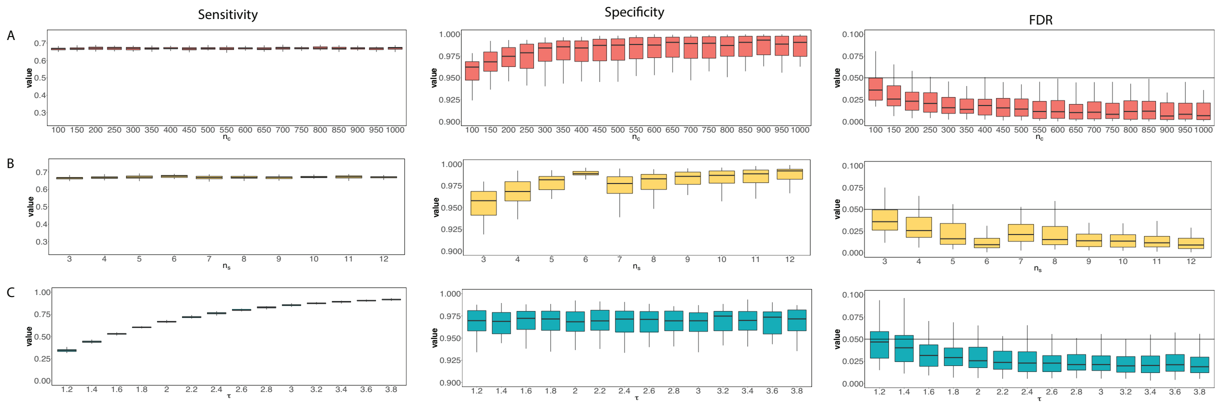

Supplementary Figure 5. Simulation results under randomly initialized $\hat{\boldsymbol{X}}$. The sensitivity, specificity, and FDR under Scenario 1 (A), 2 (B), and 3 (C).

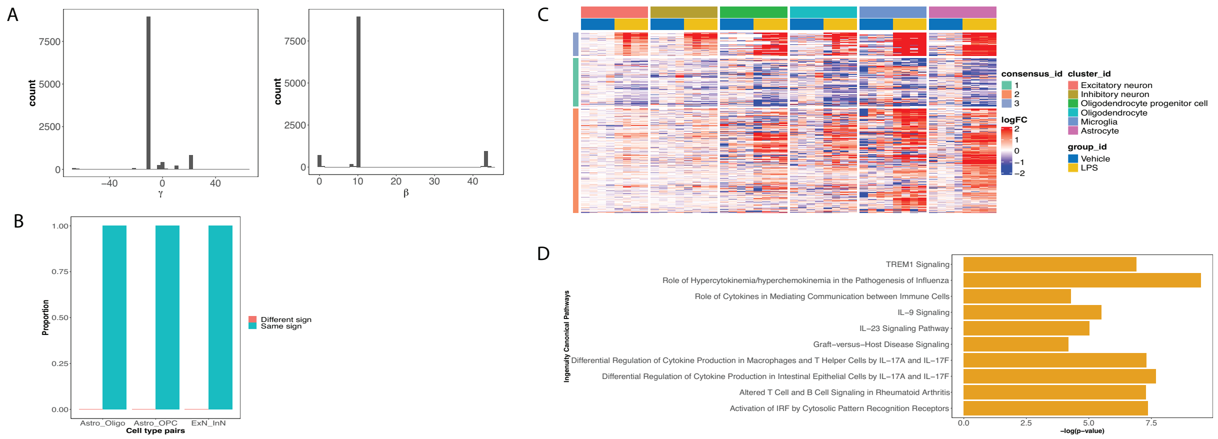

Supplementary Figure 6. edgeR-MARBLES parameter distribution, consensus clustering and pathway analysis of the mouse DE genes. (A) Histograms of the estimated model parameter γ (top) and β (bottom) across all genes. (B) Proportion of logFC of the same sign or different signs between similar cell types for DE genes identified by MARBLES. (C) M3C clustering results of the union of the DE genes across cell types. Each row represents a gene, and the color of each row shows the cell-type-specific pseudobulk expression normalized to the mean of control samples on the log scale. (D) The IPA analysis of the genes in consensus group 3 (BH corrected p value < 0.005 and gene ratio > 0.1).

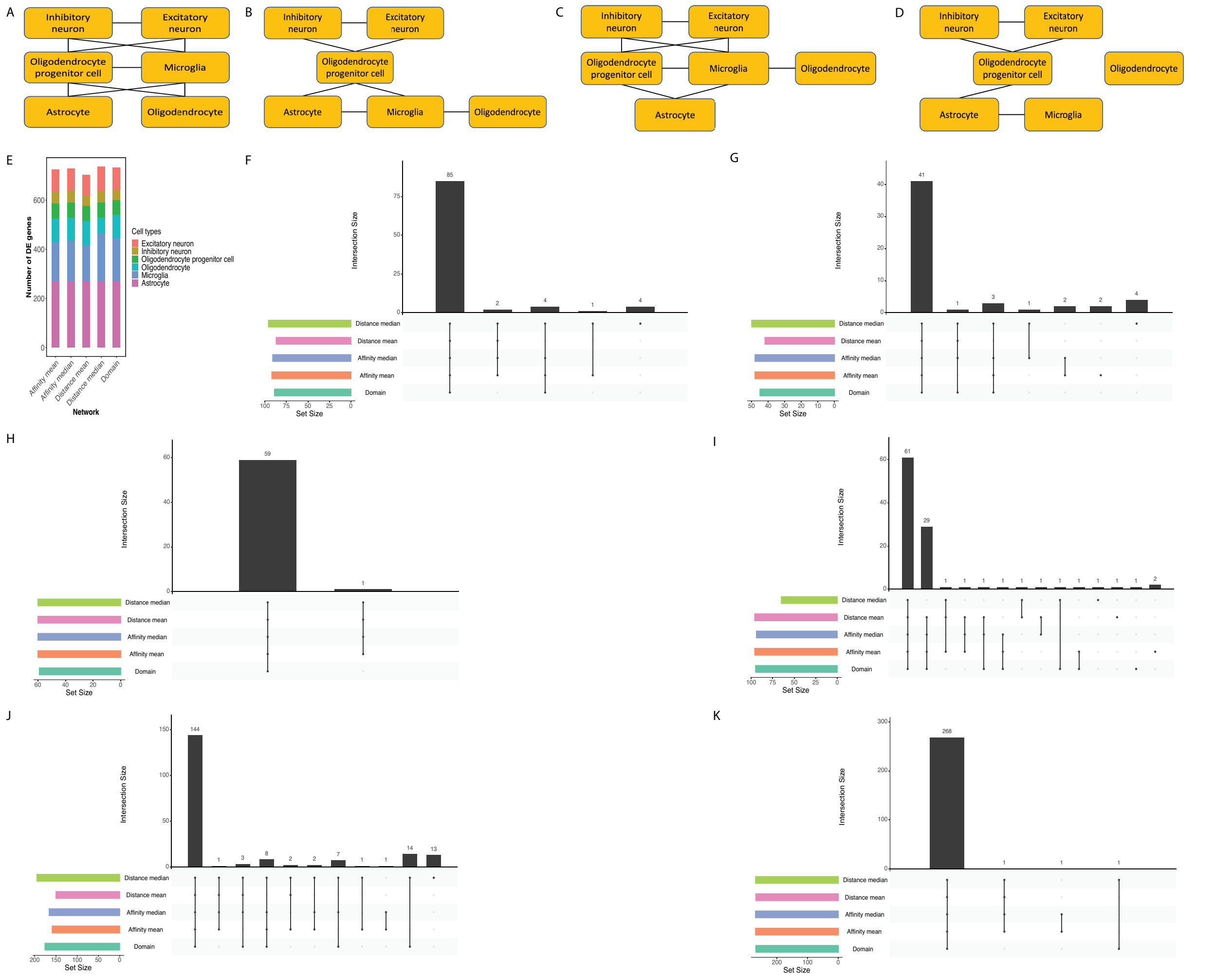

Supplementary Figure 7. edgeR-MARBLES performance under misspecified cell type networks. (A) Network based on the affinity of the mean center of each cell type cluster. (B) Network based on the affinity of the median center of each cell type cluster. (C) Network based on the distance of the mean center of each cell type cluster. (D) Network based on the distance of the median center of each cell type cluster. (E) Cell-type-specific DE genes identified by MARBLES under different networks. Domain: the main network; Affinity mean: network in (A); Affinity median: network in (B); Distance mean: network in (C); Distance median: network in (D). (F-K) Comparison of DE genes in excitatory neurons (F), inhibitory neurons (G), OPCs (H), oligodendrocytes (I), microglia (J), astrocytes (K) across different models.

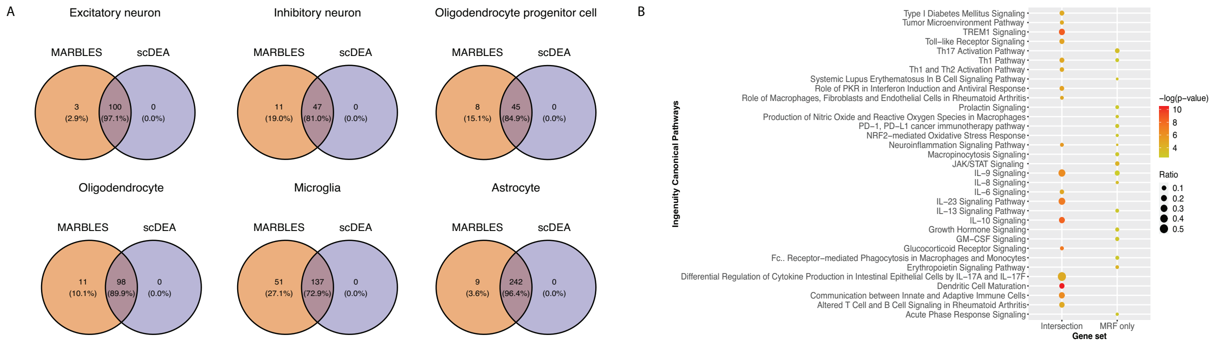

Supplementary Figure 8. scDEA-MARBLES results on the LPS mouse cortex data. (A) Venn diagrams showing the gene sets identified by scDEA alone or scDEA-MARBLES for each cell type. (B) The top IPA pathways of the DE genes in microglia identified by both scDEA and MARBLES (Intersection) or MARBLES only (MRF only).

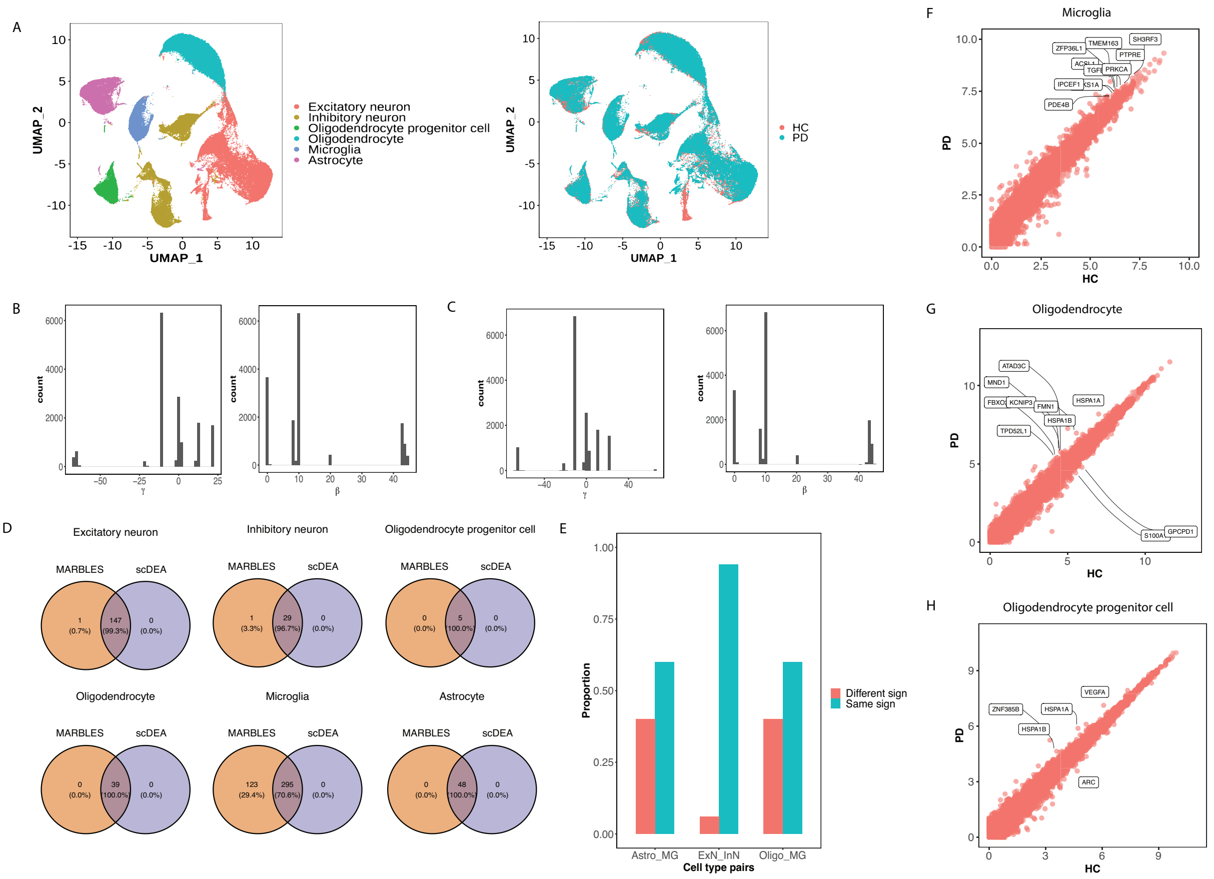

Supplementary Figure 9. Results from PD human data analysis. (A) The UMAP plot of the PD dataset colored by cell type (left) and treatment condition (right). (B-C) Histograms of the estimated model parameter γ and β across all genes for edgeR-MARBLES model (B) and scDEA-MARBLES model (C). (D) Venn diagrams showing the gene sets identified by scDEA alone or scDEA-MARBLES for each cell type. (E) Proportion of logFC of the same sign or different signs between similar cell types for DE genes identified by MARBLES. (F-H) Scatter plots of the pseudobulk-level mean expression of each gene for PD and HC in microglia (F), oligodendrocytes (G), and OPCs (H). Top 10 DE genes based on mean expression value of the pseudobulk data in log scale are shown for each cell type.
